# Prediction and identification of immune genes related to the prognosis of patients with colon adenocarcinoma and its mechanisms

**DOI:** 10.1186/s12957-020-01921-9

**Published:** 2020-06-29

**Authors:** Sihan Chen, G. D. Cao, Wu Wei, Lu Yida, He Xiaobo, Yang Lei, Chen Ke, Bo Chen, Mao Ming Xiong

**Affiliations:** Department of General Surgery, The First Affiliated Hospital of Anhui Medical University, Aahui, China

**Keywords:** Bioinformatics analysis, Prognosis, Colon adenocarcinoma, Immune genes

## Abstract

**Background:**

Colon adenocarcinoma (COAD) is a gastrointestinal tumor with a high degree of malignancy. Its deterioration process is closely related to the tumor microenvironment, and transcription factors (TF) play a regulatory role in this process. Currently, there is a lack of exploration between the genes related to the COAD tumor microenvironment and the survival prognosis of patients. Models composed of multiple genes usually predict the survival prognosis of patients more accurately than single genes. We can analyze the multigene models that can predict the prognosis of COAD from the current database.

**Methods:**

The limma package of the R programming language is used for gene differential expression analysis. Kaplan-Meier curve is used to analyze the relationship between the patient risk score model and survival data. The hazard model is used to analyze the relationship between the risk score and the clinical data of COAD patients. The information of immune genes and immune cells is obtained from IMMPORT database and TIMER database. Receiver operating characteristic (ROC) curve is used to judge the stability of the model.

**Results:**

We found 7 immune genes, which can built a risk score model to predict the survival prognosis of COAD. According to univariate and multivariate analysis, the risk score can be used as an independent predictor. The content of some immune microenvironment cells will also increase as the risk score increases.

**Conclusions:**

We found 7 immune genes, such as SLC10A2 (solute carrier family 10 member 2), CXCL3 (C-X-C motif chemokine ligand 3), IGHV5-51 (immunoglobulin heavy variable 5-51), INHBA (inhibin subunit beta A), STC1 (stanniocalcin 1), UCN (urocortin), and OXTR (oxytocin receptor), can constitute a model for predicting the prognosis of COAD. They may provide potential therapeutic targets for clinical treatment of COAD.

## Background

Colon adenocarcinoma (COAD) is a type of malignant tumor of the digestive tract, which can be subdivided into right COAD and left COAD according to location. According to a WHO report in 2018, COAD is the third most common adenocarcinoma worldwide, and 1.8 million COAD cases were diagnosed in 2018 (10% of all tumors). Adenocarcinoma of the colon is relatively common in both men and women. There were 881,000 patients who died of COAD in 2018 [1–4]. Recently, many studies have shown that the immune microenvironment plays an important role in the process of tumors [5], such as the CCR (cinnamoyl-CoA reductase) family and the CCL (CCR-like protein) famil y[6], and transcription factors can regulate this process [7, 8], but the research on the immune microenvironment in the field of COAD still needs further exploration. Compared with the single genes predicting the prognosis of cancer patients, multigene models can more accurately predict the prognosis of cancer patients, so the building of a multigene model related to the tumor microenvironment has become the focus of this research [9].

With the development of various network databases [10], such as clinical database TCGA (https://portal.gdc.cancer.gov/) [11], immune gene database IMMPORT (https://www.immport.org/shared/home) [12], TIMER (https://cistrome.shinyapps.io/timer/) [13], and transcription factor database Cistrome (http://www.cistrome.org/) [14], we can find the immune genes related to the prognosis of COAD through data analysis methods such as limma package (http://www.bioconductor.org/packages/release/bioc/html/limma.html) [15]. Cox regression analysis is used to build a risk score model of immune genes related to prognosis. Seven immune genes building this model are SLC10A2 (solute carrier family 10 member 2), CXCL3 (C-X-C motif chemokine ligand 3), IGHV5-51 (immunoglobulin heavy variable 5-51), INHBA (inhibin subunit beta A), STC1 (stanniocalcin 1), UCN (urocortin), and OXTR (oxytocin receptor). We define the sample with the highest risk score of 50% as the high-risk group and the sample with the lowest risk score of 50% as the low-risk group. Subsequent studies will combine relevant clinical data to further compare the differences between the two groups.

The result of this study is we built a multigene model related to the immune microenvironment, which can predict the prognosis of COAD patients. These seven immune genes may provide potential therapeutic targets for clinical treatment of COAD.

## Methods

### Gets the relevant data from the network database

Obtaining COAD gene expression data and clinical data from the TCGA (https://portal.gdc.cancer.gov/) database, immune gene names were obtained from the IMMPORT (https://www.immport.org/shared/home) database, and immune genes were screened from the downloaded data. Transcription factor data from the Cistrome (http://www.cistrome.org/) database and tumor microenvironment-related gene infiltration data were obtained from the TIMER (https://cistrome.shinyapps.io/timer/) database.

### Acquisition of differentially expressed genes of interest

Using the R script (Gene.diff.R) to obtain differentially expressed genes of interest, then, the differential expression analysis of immune genes uses R script (immuneGene.immuneDiff.R), and the screening condition is LogFC (log fold change) > 2 and FDR (false discover rate) < 0.05. Then, differentially expressed transcription factors were analyzed using R script (immuneGene.TFdiff.R), and the screening conditions were LogFC > 1 and *P* < 0.05.

### Obtain immune genes related to the survival prognosis of COAD patients

Univariate Cox regression analysis was performed on the differentially expressed immune genes obtained by the above method and the clinical data of COAD patients, and then, the immune genes related to the prognosis of COAD patients were obtained. The screening conditions are *P* < 0.01 and hazard ratio ≠ 1(immuneGene.uniCox.R).

### Calculation of risk score and independent prognostic analysis

First, through univariate Cox regression analysis, the immune genes related to the prognosis of COAD are obtained and then through multivariate Cox regression to find the immune genes that can build the risk score model (immuneGene.multiCox.R). Risk score = ExpmRNA1 × coefmRNA1 + ExprmRNA2 × coefmRNA2 +···+ ExpmRNAn × coefmRNA. “Exp” indicates the expression level of the gene; “coef” indicates the correlation coefficient of the gene. Finally, we combined clinical data and used immuneGene.uniIndep.R and immuneGene.multiIndep.R for univariate independent prognostic analysis and multivariate independent prognostic analysis.

### Immune genes will draw the interaction network of transcription factors

Mapping the interaction network of immune genes and transcription factors using R script (immuneGene.TFcor.R) and Cytoscape [16], the screening conditions are Cor = 0.4 and *P* = 0.01.

### Analysis of clinical correlation of 7 genes (building of risk scoring model)

We correlate the risk score with clinical data and analyze and make the corresponding models. Building of Kaplan-Meier curve (K-M) uses R script (immuneGene.survial.R); the building of receiver operating characteristic (ROC) model uses R script (immuneGene.ROC.R); the building of risk curve uses R script (immuneGene.riskPlot.R); clinical correlation was built using R script (immuneGene.clincialCor.R), and the immune cell correlation graph was built using R script (immuneGene.immuneCor.R). The K-M curve is used to express the relationship between risk scores and patient survival data. *P* < 0.05 is considered statistically significant. The ROC curve is used to indicate the sensitivity of the model. 0.5–0.7 means the sensitivity is acceptable, 0.7–0.9 means the sensitivity is good, and > 0.9 means the sensitivity is excellent.

### Correlation analysis between samples and tumor microenvironment

Twenty samples from the top 10 and the bottom 10 of the risk score were selected to analyze the cells composed of the tumor microenvironment. The TIMER database was used for this analysis and draw (Fig. 9). Figure 8 is drawn using R script (immuneGene.immuneCor.R).

## Result

### Acquisition of immune DEGs and differentially expressed transcription factors

**Fig. 1:**
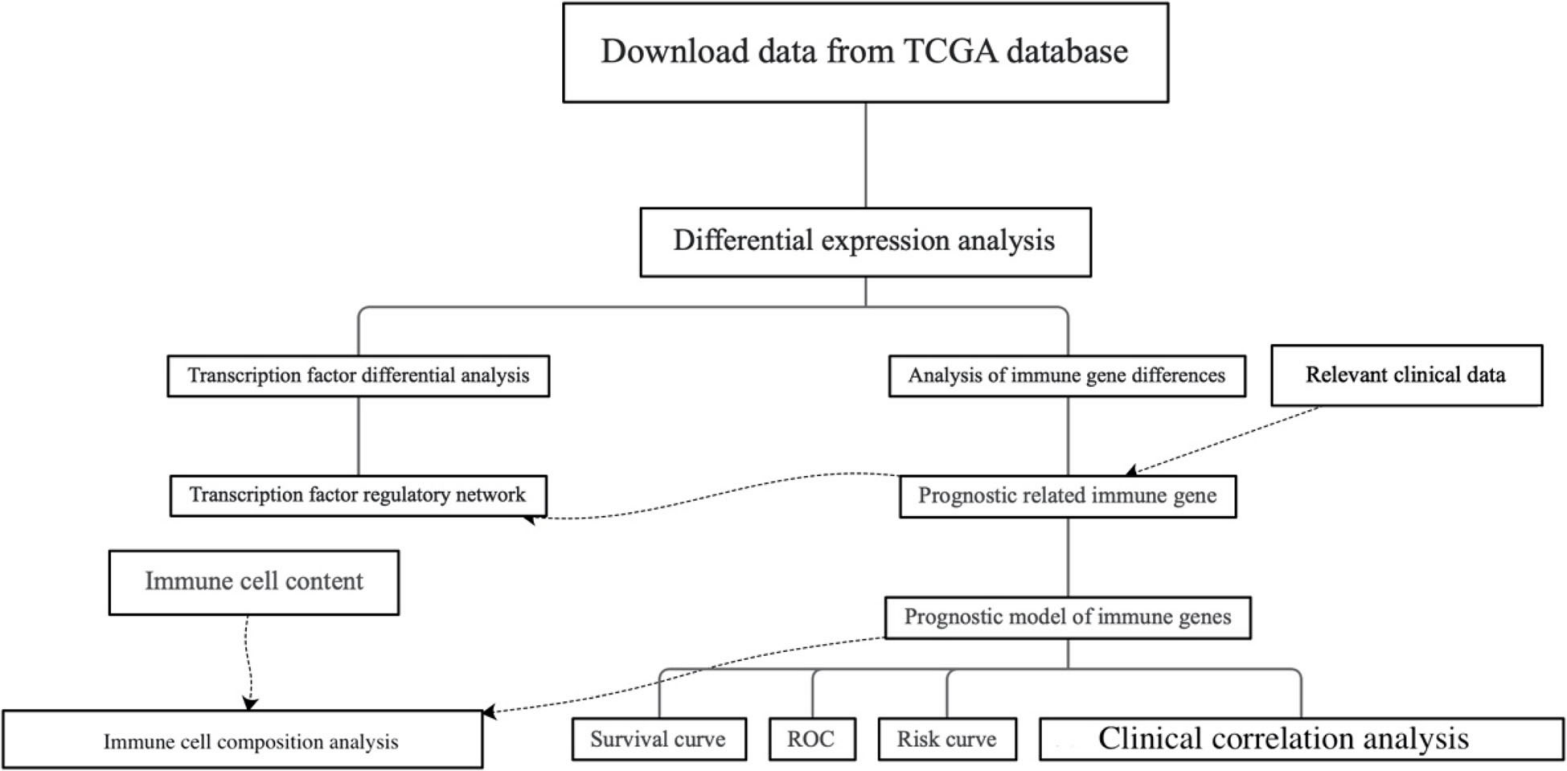
Flow chart of this study

**Fig. 2:**
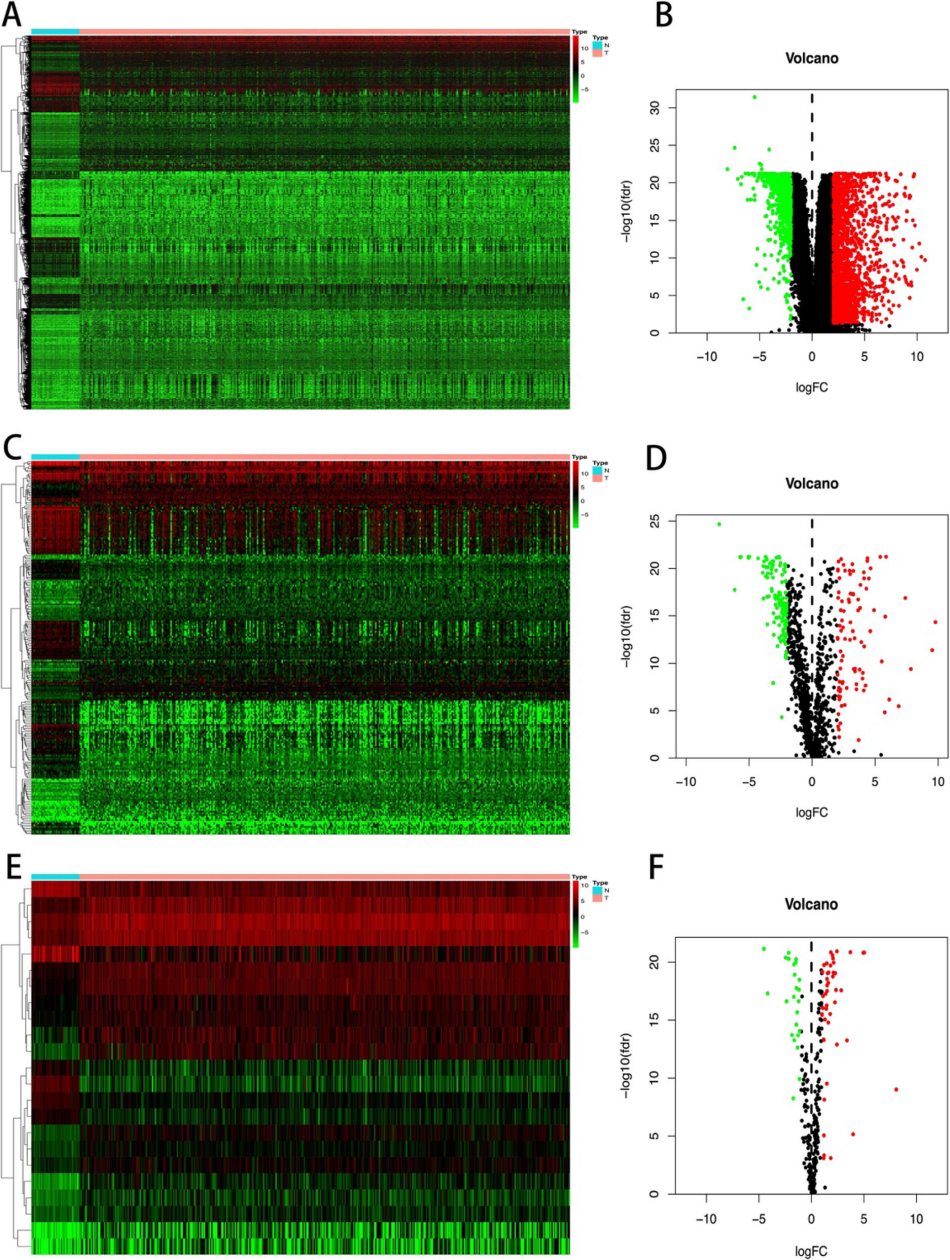
**a** Heat map of genes expression. **b** Volcano map of genes expression. **c** Heat map of immune genes expression. **d** Volcano map of immune genes expression. **e** Heat map of transcription factors expression. **f** Volcano map of transcription factors expression

The research team downloaded clinical data and gene expression data of 385 COAD patients from the TCGA (https://portal.gdc.cancer.gov/) database and obtained differentially expressed genes (DEGs) through screening (screening conditions: LogFC > 2 and FDR < 0.05) (Figs. 1 and 2a, b). The IMMPORT (https://www.immport.org/shared/home) database contains the names of a large number of immune genes. We obtain the differentially expressed immune genes through the intersection of the immune gene names and DEGs (Fig. 2c, d). The transcription factor names were obtained from the Cistrome (http://www.cistrome.org/) database and then screened for eligible transcription factors from DEGs. The screening conditions for transcription factors are LogFC > 1 and *P* < 0.05. The volcano and heat maps were drawn (Fig. 2e, f).

### Building of the prognostic-related immune gene model and the interaction network of prognostic-related immune genes and transcription factors

**Fig. 3:**
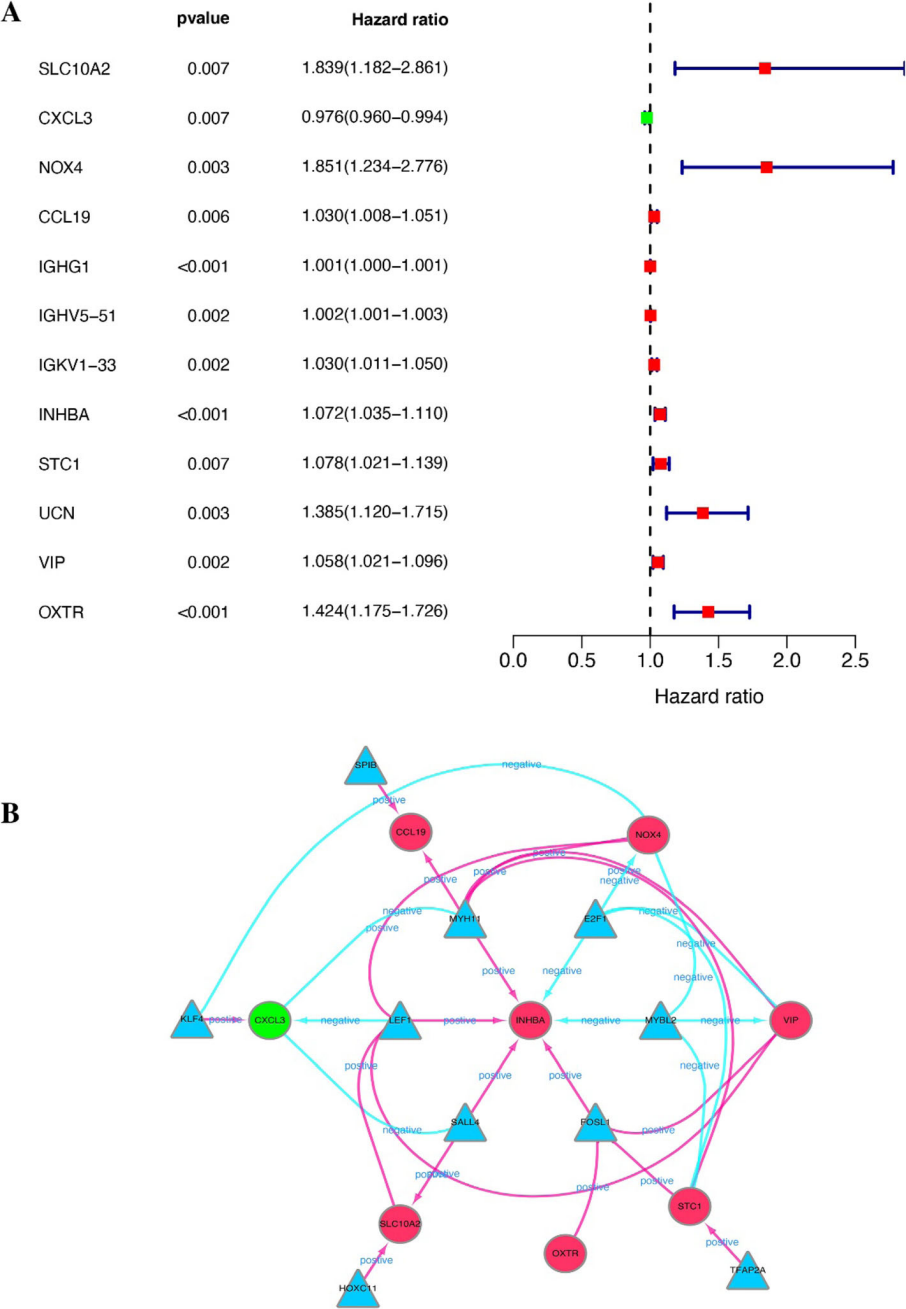
**a** Forest plot of 12 immune genes associated with survival prognosis in patients with COAD. **b** Regulatory network of immune genes and transcription factors related to the prognosis of COAD. The circles represent immune genes (green represents a downward adjustment, and red represents an upward adjustment), and the triangles represent transcription factors

Univariate Cox regression analysis was used to study the differentially expressed immune genes related to survival prognosis. The results showed that there are 12 immune genes that are closely related to the prognosis of COAD (Fig. 3a). Cor = 0.4 and *P* = 0.01 screening criteria were used to establish the interaction between immune genes and transcription factors, and network diagrams were made (Fig. 3b). Details of the regulatory relationship between transcription factors and the immune genes associated with COAD prognosis are shown in Table 1. The results showed that 8 immune genes are closely related to the regulation of transcription factors and belong to positive regulation.

**Table 1:** Details of the regulatory relationship between transcription factors and immune genes

TF	Immune gene	Cor	*P* value	Regulation
E2F1	NOX4	− 5.63E−01	1.14E−06	Negative
	INHBA	− 5.26E−01	3.04E−05	Negative
	STC1	− 4.67E−01	2.17E−03	Negative
	VIP	− 4.71E−01	1.75E−03	Negative
FOSL1	INHBA	4.67E−01	2.16E−03	Positive
	STC1	4.67E−01	2.18E−03	Positive
	VIP	4.57E−01	2.01E−06	Positive
	OXTR	4.85E−01	7.00E−04	Positive
HOXC11	SLC10A2	9.34E−01	3.98E−10	Positive
KLF4	CXCL3	5.15E−01	7.63E−05	Positive
	NOX4	− 4.96E−01	3.09E−04	Negative
LEF1	SLC10A2	4.87E−01	5.95E−04	Positive
	CXCL3	− 4.56E−01	4.22E−03	Negative
	NOX4	4.82E−01	8.60E−04	Positive
	INHBA	4.84E−01	7.51E−04	Positive
	VIP	4.65E−01	2.56E−03	Positive
MYBL2	NOX4	− 5.45E−01	5.71E−06	Negative
	INHBA	− 5.31E−01	2.01E−05	Negative
	STC1	− 4.53E+01	4.94E−03	Negative
	VIP	− 4.58E−01	3.75E−03	Negative
MYH11	CXCL3	− 4.58E−01	3.80E−03	Negative
	NOX4	4.55E−01	4.44E−03	Positive
	CCL19	6.40E−01	1.84E−10	Positive
	INHBA	4.80E−01	9.71E−04	Positive
	STC1	4.68E−01	2.04E−03	Positive
	VIP	7.22E−01	5.91E−20	Positive
SALL4	SLC10A2	6.22E−01	1.66E−09	Positive
	CXCL3	− 4.84E−01	7.37E−04	Negative
	INHBA	4.70E−01	1.81E−03	Positive
SPIB	CCL19	4.77E−01	1.17E−03	Positive
TFAP2A	STC1	4.49E−01	6.26E−03	Positive

### Calculation of immune gene risk score and building of survival prognosis model

**Fig. 4:**
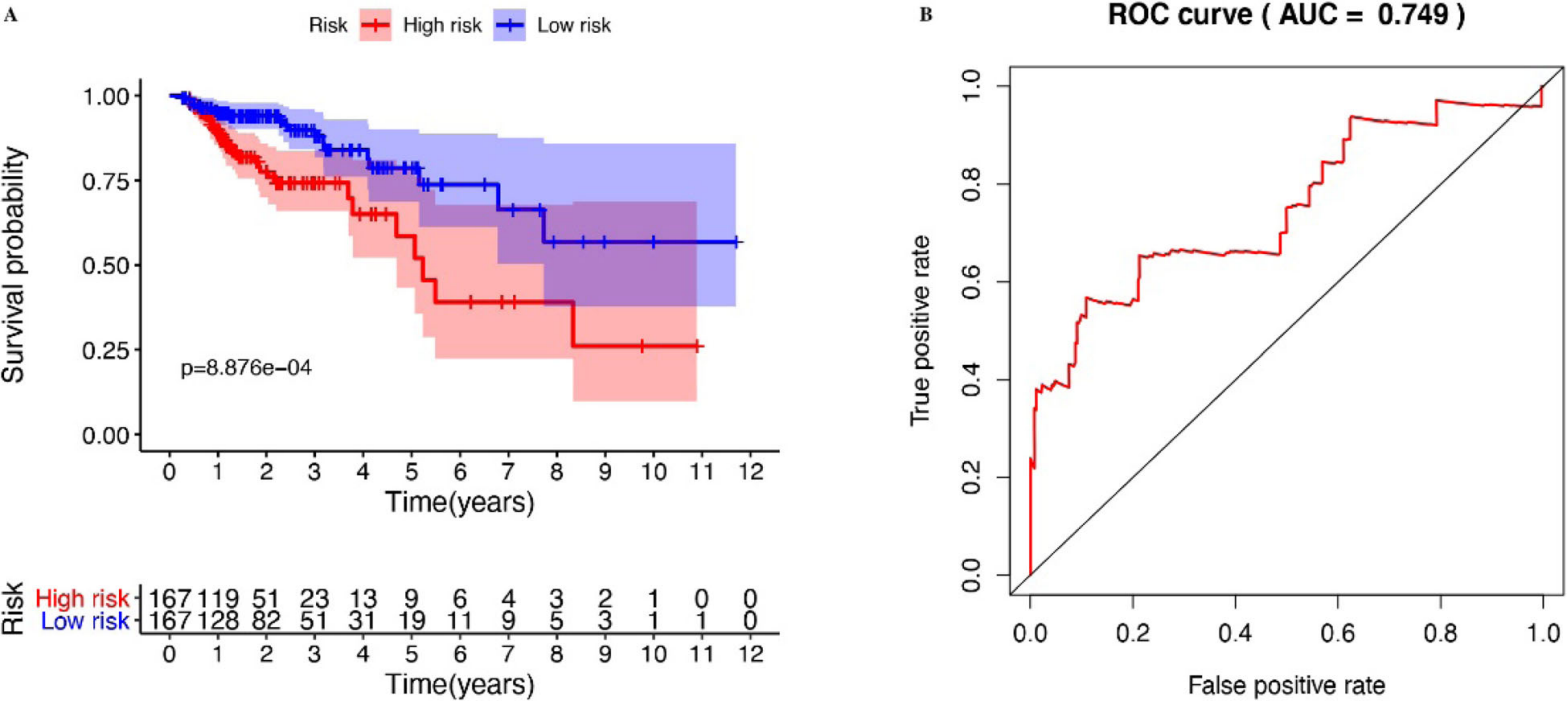
**a** Kaplan-Meier survival curve of high-risk group and low-risk group. **b** ROC curve of survival prognosis model (0.5–0.7 means the sensitivity is acceptable, 0.7-0.9 means the sensitivity is good, and > 0.9 means the sensitivity is excellent)

The 12 immune genes (Fig. 3a) related to the survival prognosis of COAD patients obtained by univariate Cox analysis were included. Then, using multivariate Cox regression analysis, the screening conditions were *P* < 0.05 and hazard ratio (HR) ≠ 1, and 7 immune genes were eligible and included for further calculation of risk score. We define the sample with the top 50% of the risk score as the high-risk group and the sample with the bottom 50% of the risk score as the low-risk group. Subsequent studies will further compare the differences between the two groups. Among the immune genes related to survival prognosis, 7 immune genes are closely related to the composition of risk score, which are SLC10A2, CXCL3, IGHV5-51, INHBA, STC1, UCN, and OXTR (Table 2); this is also the key immune gene that we will study later. According to the median risk score, the risk score is divided into two groups. Survival and surmiser packages in R were used to correlate risk score with survival prognosis and draw the Kaplan-Meier survival curves. The results showed that *P* = 8.876e−04. The survival prognosis of the high-risk group was significantly worse than that of the low-expression group (Fig. 4a). The survivalROC package of R language is used to draw the ROC curve. The results show that the AUC of the ROC curve = 0.749 (Fig. 4b). Detailed data on the survival rates of high- and low-risk patients are shown in Tables 3 and 4.

**Table 2:** Details of the seven immune genes used to build the risk score model

Id	Coef	HR	HR.95L	HR.95H	*P* value
SLC10A2	0.65	1.916	1.203	3.05	0.006
CXCL3	− 0.019	0.981	0.964	0.998	0.033
IGHV5-51	0.002	1.002	1	1.003	0.005
INHBA	0.046	1.047	1.003	1.093	0.038
STC1	0.058	1.059	0.991	1.133	0.092
UCN	0.405	1.499	1.198	1.876	0
OXTR	0.229	1.258	0.996	1.588	0.054

**Table 3:** Detailed data for high risk survival analysis

Time (year)	n.Risk	n.Event	Survival (%)	Std.err	Lower 95% CI	Upper 95% CI
0.266	167	1	0.994	0.00597	0.9824	1
0.419	163	1	0.988	0.0085	0.9714	1
0.427	160	1	0.982	0.01045	0.9615	1
0.436	159	1	0.976	0.01207	0.9522	1
0.471	158	1	0.969	0.01348	0.9433	0.996
0.515	154	1	0.963	0.01479	0.9345	0.993
0.586	150	1	0.957	0.01602	0.9258	0.989
0.625	149	1	0.95	0.01715	0.9172	0.984
0.718	142	1	0.944	0.01829	0.9084	0.98
0.795	136	1	0.937	0.01943	0.8993	0.975
0.827	134	1	0.93	0.0205	0.8903	0.971
0.838	131	2	0.915	0.02251	0.8724	0.961
0.907	127	1	0.908	0.02346	0.8634	0.955
0.926	123	1	0.901	0.0244	0.8543	0.95
1.008	117	1	0.893	0.02538	0.8448	0.944
1.049	112	1	0.885	0.02638	0.835	0.938
1.085	108	1	0.877	0.02738	0.8249	0.932
1.104	106	1	0.869	0.02834	0.8149	0.926
1.162	101	1	0.86	0.02934	0.8045	0.92
1.167	99	1	0.851	0.0303	0.7941	0.913
1.293	88	1	0.842	0.03146	0.7823	0.906
1.359	81	1	0.831	0.03275	0.7696	0.898
1.4	77	1	0.821	0.03405	0.7565	0.89
1.762	57	1	0.806	0.03637	0.7379	0.881
1.836	53	1	0.791	0.03874	0.7186	0.871
1.868	52	1	0.776	0.04087	0.6996	0.86
2.036	49	1	0.76	0.04299	0.6801	0.849
2.205	46	1	0.743	0.04512	0.66	0.837
3.693	16	1	0.697	0.06175	0.5858	0.829
3.784	15	1	0.65	0.07305	0.5219	0.811
4.688	10	1	0.585	0.09017	0.4329	0.792
5.066	9	1	0.52	0.10092	0.3558	0.761
5.233	8	1	0.455	0.10724	0.287	0.722
5.488	7	1	0.39	0.10989	0.2248	0.678
8.334	3	1	0.26	0.12903	0.0984	0.688

**Table 4:** Detailed data for low risk survival analysis

Time (year)	n.Risk	n.Event	Survival (%)	Std.err	Lower 95% CI	Upper 95% CI
0.247	167	1	0.994	0.00597	0.982	1
0.4	161	1	0.988	0.00855	0.971	1
0.419	159	2	0.975	0.01214	0.952	1
0.564	154	1	0.969	0.01362	0.943	0.996
0.663	148	1	0.963	0.01502	0.934	0.992
0.918	138	1	0.956	0.01645	0.924	0.988
0.978	133	1	0.948	0.01783	0.914	0.984
1.211	114	1	0.94	0.01951	0.903	0.979
2.252	71	1	0.927	0.0233	0.882	0.974
2.351	69	1	0.913	0.02655	0.863	0.967
2.463	66	1	0.9	0.02954	0.843	0.959
2.997	52	1	0.882	0.03366	0.819	0.951
3.173	43	1	0.862	0.03863	0.789	0.941
3.184	42	1	0.841	0.04281	0.761	0.929
4.09	31	1	0.814	0.04928	0.723	0.917
4.118	30	1	0.787	0.0546	0.687	0.902
5.153	16	1	0.738	0.06992	0.613	0.888
6.781	10	1	0.664	0.09412	0.503	0.877
7.729	7	1	0.569	0.11925	0.377	0.858

### Immune gene risk curve mapping and independent prognostic analysis

**Fig. 5:**
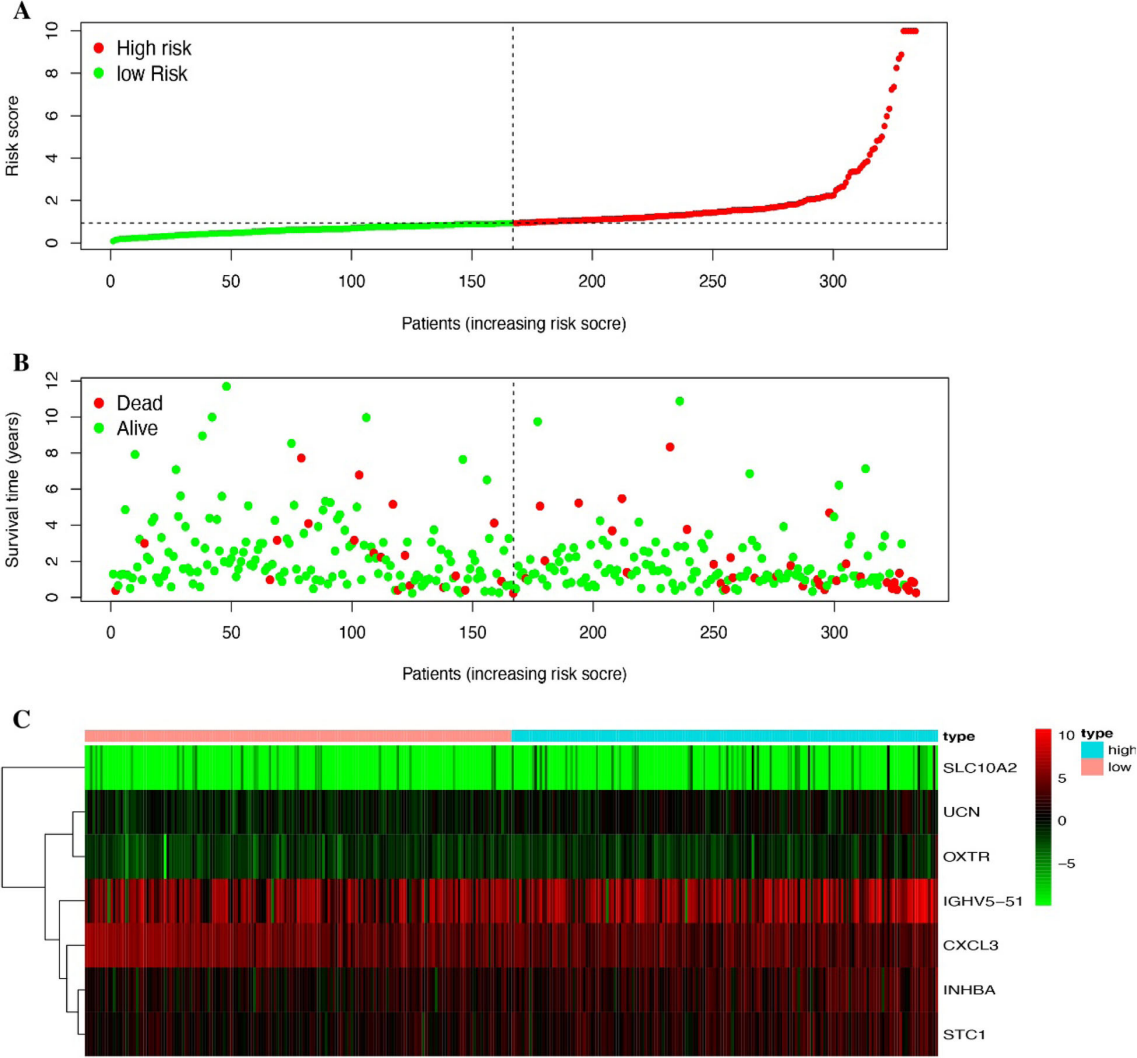
**a** Risk curve of risk score growth trend. **b** Diagram of the relationship between risk score and patient survival time. **c** Building of a heat map of immune genes in the risk score model

**Fig. 6:**
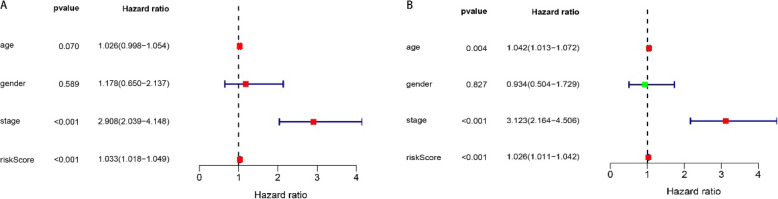
**a** Forest plot of univariate Cox regression analysis between immune genes and clinical data constituting the risk score model. **b** Forest plot of multivariate Cox regression analysis between immune genes and clinical data that constitute the risk score model

Using R language related codes to draw related pictures of the risk curve, the results showed that with the gradual increase of the immune gene risk value, the survival time of patients gradually decreased (Fig. 5a, b). Heat map of related immune gene expression is shown in Fig. 5c. Univariate independent prognostic analysis showed that the hazard ratio of risk score was 1.033 (1.018–1.049) and *P* < 0.001. Multivariate independent prognostic analysis showed that hazard ratio of risk score was 1.026 (1.011–1.042) and *P* < 0.001 (Fig. 6a, b). Risk scores are clinically and statistically significant.

### Correlation analysis of immune genes and clinical data

**Fig. 7:**
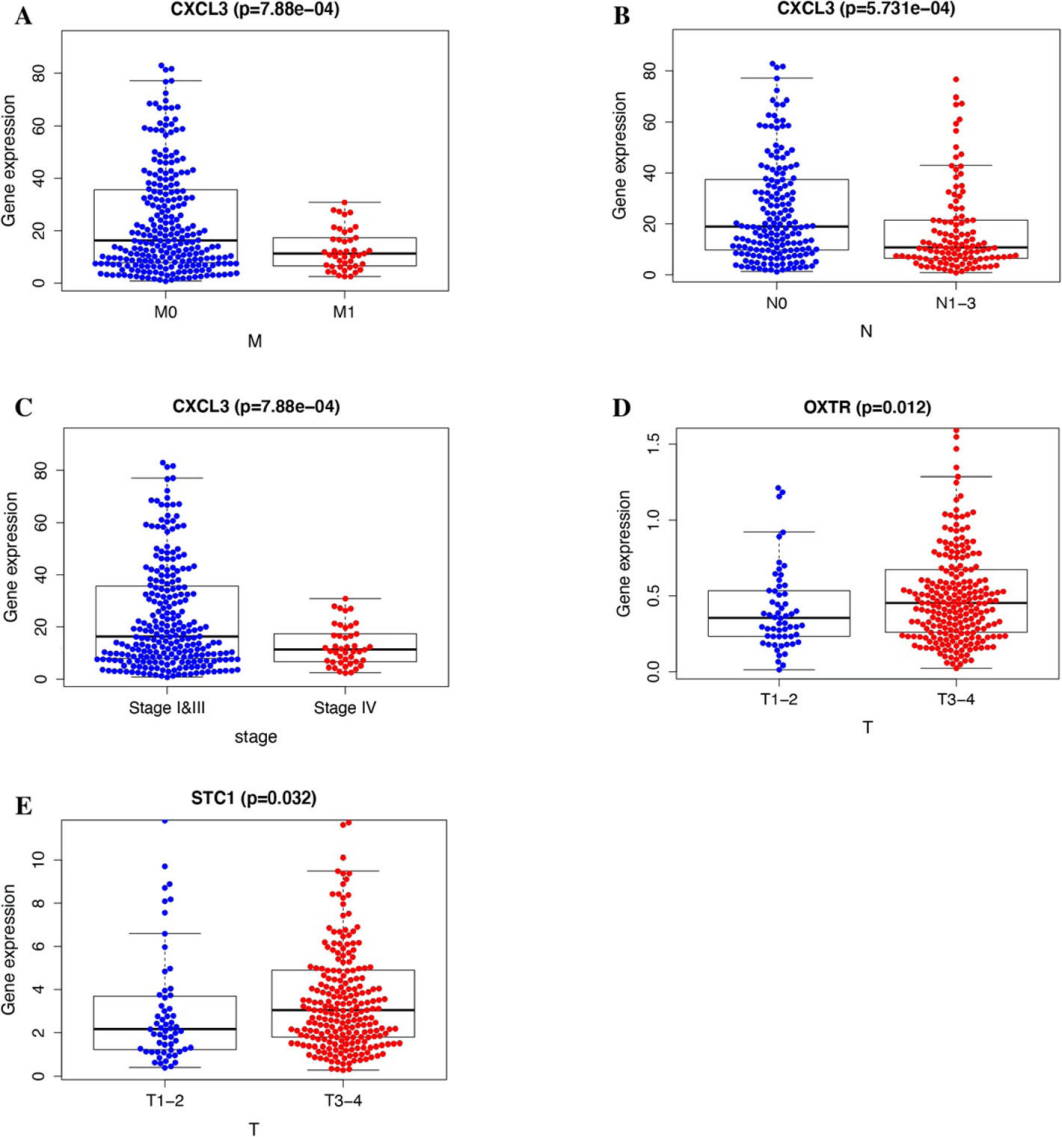
Correlation analysis of genes (building a risk score model) and clinical data. **a** Correlation analysis between CXCL3 and M. **b** Correlation analysis between CXCL3 and N. **c** Correlation analysis between CXCL3 and COAD stage. **d** Correlation analysis between OXTR and T. **e** Correlation analysis between STC1 and T. T1–2, the tumor invades the submucosa, or the tumor invades the muscularis intestinal wall; T3–T4, the tumor infiltrates the muscularis laminae and reaches the subserosa, or the tumor has penetrated the peritoneum; N0, no regional lymph node metastasis; N1–3, there is metastasis in regional lymph nodes; M0, the tumor has no distant metastasis; M1, the tumor has distant metastasis; stage, the stage of colon cancer. Stages I, II, and III are early colon cancer, and IV is advanced colon cancer

We analyzed the correlation between the 7 immune genes that make up the immune score and clinical data, using the R language beeswarm package. The results showed that there were statistically significant correlations between seven immune genes and clinical data, namely, CXCL3, OXTR, and STC1 (Fig. 7). Among them, the expressions of CXCL3 were statistically significant in correlation with stage, while the expressions of OXTR and STC1 were statistically significant in correlation with T. CXCL3 also has significant difference in N and M. In T1–2, the tumor invades the submucosa, or the tumor invades the muscularis intestinal wall; in T3–T4, the tumor infiltrates the muscularis laminae and reaches the subserosa, or the tumor has penetrated the peritoneum; in N0, no regional lymph node metastasis; in N1–3, there is metastasis in regional lymph nodes; in M0, the tumor has no distant metastasis; in M1, the tumor has distant metastasis. Stages I, II, and III are early colon cancer, and stage IV is advanced colon cancer.

### Correlation analysis of risk score and tumor microenvironment cells

**Fig. 8:**
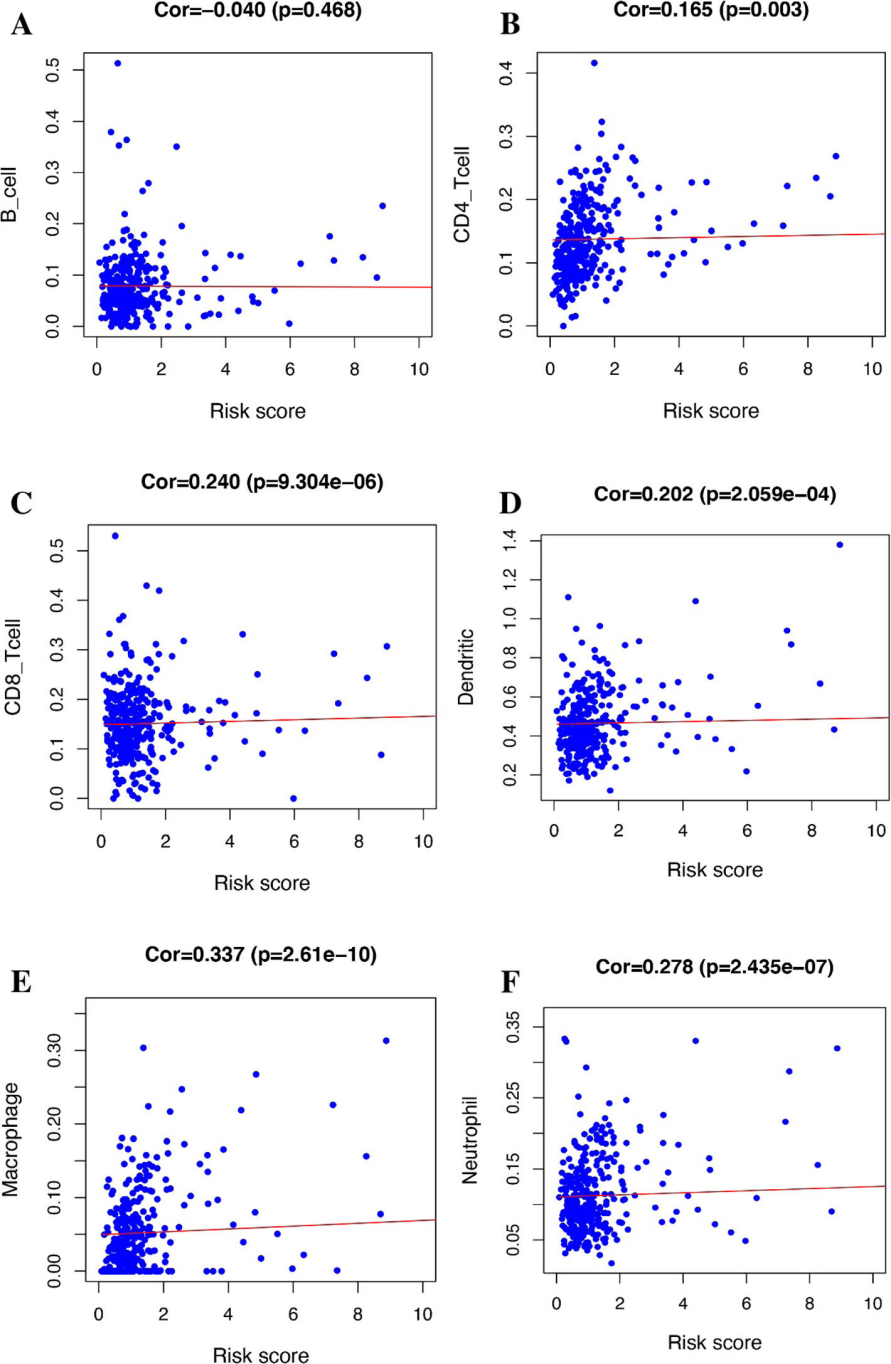
Correlation analysis between the expression of immune microenvironment cells and risk score. **a** Correlation analysis between B cells and risk score. **b** Correlation analysis between CD4 cells and risk score. **c** Correlation analysis between CD8 cells and risk score. **d** Correlation analysis between dendritic cells and risk score. **e** Correlation analysis between macrophage cells correlation and risk score. **f** Correlation analysis between neutrophil cells and risk score

Correlation analysis was performed between the risk score assessed by our research and immune microenvironment genes, and the results showed that in CD4, CD8, dendritic, macrophage, and neutrophil cells, as the risk score increased, the expression levels of these genes became upward. And it has statistical significance *P* < 0.05. Correlation analysis of our risk value model with some widely recognized genes that constitute the immune microenvironment showed that CD4, CD8, dendritic, macrophage, and neutrophil cells were positively correlated with the risk score model. As the risk score increases, so does the expression of these genes (Fig. 8).

### Correlation between risk score model and tumor microenvironment

**Fig. 9:**
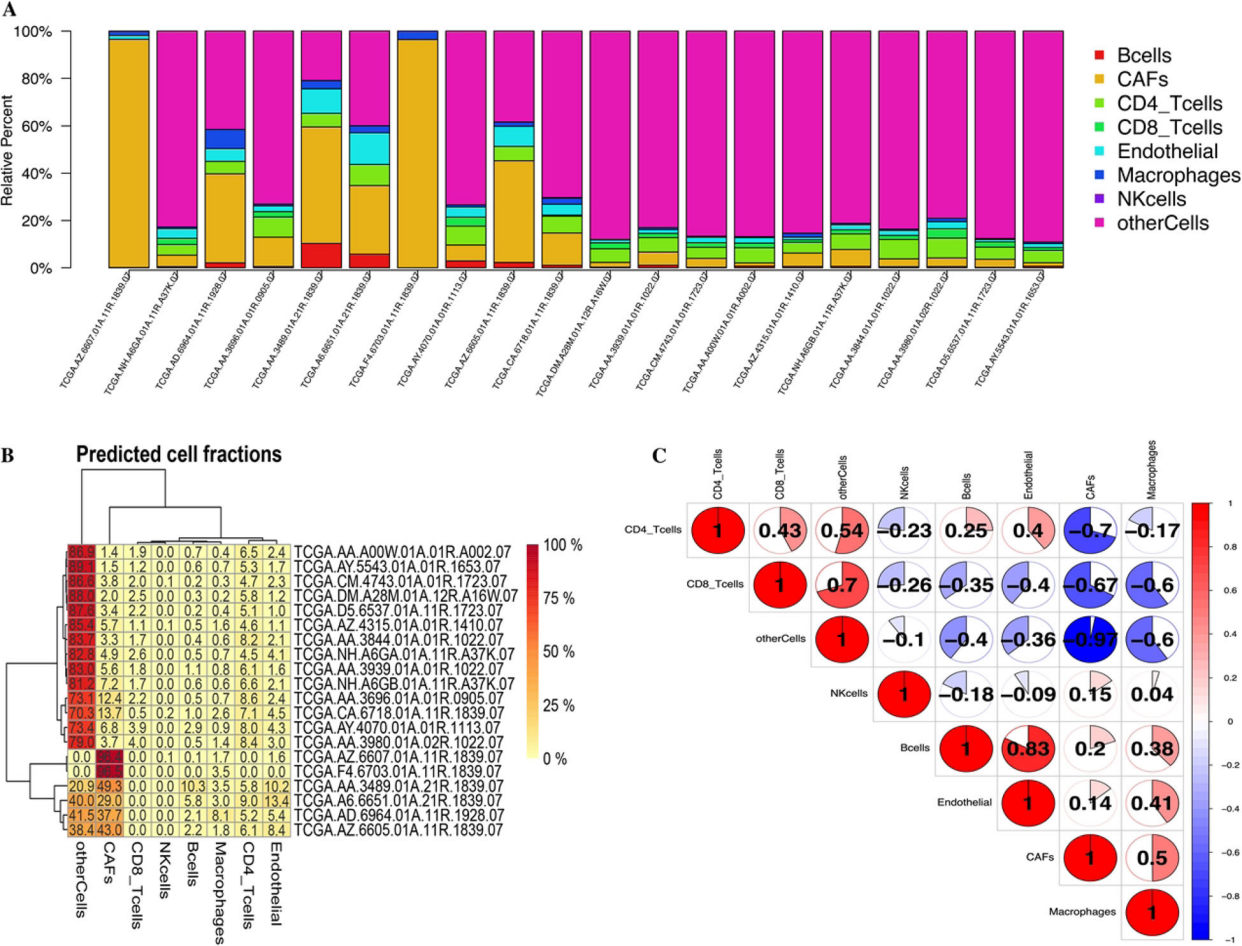
**a** Comparison of immune cell differences between 10 groups of high risk score samples and 10 groups of low risk score samples, each color represents a different cell type. **b** The expression of immune cells differs between 10 groups of high risk score samples and 10 groups of low risk score samples. **c** Immune cell correlation matrix, the positive correlation is red, and the negative correlation is blue

To evaluate the difference in immune cell content between samples with high risk score and samples with low risk score, we selected 20 sets of samples, which were selected from the 10 samples with the highest risk score and the 10 samples with the lowest risk score, through the EPIC database. Calculate the difference in the amount of their direct immune cells, the results showed that in 10 samples with high risk score, the content of cancer-associated fibroblasts (CAFs) cells was significantly higher than that with 10 samples with low risk score. From this, we can know that CAFs cells play an important role in risk score (Fig. 9).

## Discussion

The flow of this study is shown in Fig. 1. We searched the TCGA (https://portal.gdc.cancer.gov/) database for 385 cases of COAD and downloaded them. The clinical data and gene expression data in the download data were integrated, and the limma package of R language was used to extract differentially expressed genes (DEGs). Using the immune gene names provided by IMMPORT (https://www.immport.org/shared/home), we can easily screen out the differential gene immune genes from DEGs. In the same way, we download the names of transcription factors from the Cistrome (http://www.cistrome.org/) database and screen out the differentially expressed transcription factors from DEGs (screening conditions: LogFC > 2 and FDR < 0.05). We then conduct further analysis of differentially expressed immune genes (screening conditions: LogFC > 2 and FDR < 0.05) and differentially expressed transcription factors (screening conditions: LogFC > 1 and FDR < 0.05). The differentially expressed immune genes were analyzed by univariate Cox regression using the survival package of R language and clinical data to obtain immune genes related to prognosis. Prognostic-related immune genes are as follows: SLC10A2, CXCL3, NOX4, CCL19, IGHG1, IGHV5-51, IGKV1-33, INHBA, STC1, UCN, VIP, and OXTR (Fig. 3a). We performed an interaction network analysis of prognostic-associated immune genes and differentially expressed transcription factors, and the results are shown in Fig. 3b. Analysis results show that the regulatory network functions are mainly concentrated in optic vesicle morphogenesis and regulation of leukocyte adhesion to arterial endothelial cells, but more specific mechanisms need further study [17].

We excluded samples with a survival time of less than 90 days from the downloaded clinical data and assessed the survival prognosis by the level of risk score. The results showed that patients with high risk score had significantly worse survival prognosis than patients with low risk score, *P* = 8.876e−04 (Fig. 4a). The ROC curve showed that AUC = 0.749, and the risk score and prognosis model were more reliable (Fig. 4b). This allows us to group patients according to the risk scores in clinical work to predict their prognosis. According to Fig. 5 a and b, we can find that as the risk score increases, the survival time of the patient decreases. The heat map of Fig. 5c also shows that the genes that build the risk score have higher expression levels in the high risk score array. We included clinical data on COAD and the risk score evaluated in this study into the Cox regression analysis. The results showed that stage, T, M, N, and risk score were statistically significant and clinically significant in the survival prognosis of the patients in the univariate Cox regression analysis. However, in the results of multivariate Cox regression analysis, age, stage, T, and risk score have statistical significance and clinical significance. Based on the analysis of the seven genes and clinical data used to build the risk score model, the results show that the expression of CXCL3 gene in M, N, and stage is higher than that in late stage. This is likely to be related to the mechanism of the immune microenvironment [18]. Studies on the immune microenvironment have shown that some genes that build the immune microenvironment can promote tumor progression (Fig. 7). Some cells that make up the tumor microenvironment, such as B, CD4, CD8, dendritic, macrophage, and neutrophil cells, have been shown in research to be correlated with the survival prognosis of many types of tumor patients [9]. We downloaded the data of these cells through the TIMER (https://cistrome.shinyapps.io/timer/) database and performed correlation analysis with the risk score model we built. The results showed that the expression of CD4, CD8, dendritic, macrophage, and neutrophil cells increased with the increase of the risk score. This also confirms on the side that the risk score model we built has a certain predictive ability for the clinical prognosis of patients.

The formation of the tumor microenvironment is closely related to the occurrence and development of tumors [9]. By studying the cells that constitute the tumor microenvironment, we can effectively find many cells or genes that are closely related to the clinical prognosis of patients. To evaluate the difference in immune cell content between samples with high risk score and samples with low risk score, we further evaluated them in the EPIC database. We selected 20 sets of samples, which were selected from the 10 samples with the highest risk score and the 10 samples with the lowest risk score, which passed the EPIC database [19]. Calculate the difference in the amount of their direct immune cells, the results showed that in 10 samples with high risk score, the content of CAFs cells was significantly higher than that with 10 samples with low risk score. From this, we can know that CAFs cells play an important role in risk score. The related literature reports that cancer-associated fibroblasts (CAFs) are the main cell types in the tumor stromal. CAFs usually promote tumor progression by inducing cell proliferation, inflammation, blood vessel growth, and metastasis. We judged that the content of CAFs is also an important indicator to increase the risk score [20].

In this study, we built an immune gene risk score model for 385 COAD patients through correlation analysis. Through a series of analyses of the disease, it was found that the risk score is closely related to the survival prognosis of patients. In future clinical treatments, we can use the risk score model to effectively predict the survival prognosis of patients with COAD, and we can do targeted immunotherapy for 7 immune genes (SLC10A2, CXCL3, IGHV5-51, INHBA, STC1, UCN, and OXTR) that constitute the risk score to improve the prognosis of patients and improve the treatment effect.

Bile acids, especially secondary bile acids, can promote the development of colorectal cancer, and SLC10A2 can promote this process [21, 22]. CXCL3 is related to the occurrence and development of prostate cancer, colon cancer, and breast cancer. There are also reports in the literature that the effect of suppressing the development of colon cancer can be achieved by immunosuppression of CXCL3 [23–27]. INHBA has a significant relationship with the occurrence and development of gastric, esophageal, and ovarian cancers, and studies have reported that the immunosuppressive treatment of INHBA can reduce the rate of deterioration of gastric and ovarian cancers [28–30]. STC1 can promote the metastasis of colon cancer [31, 32].

## Conclusion

We download data for COAD, immune genes, and transcription factors through a series of bioinformatics databases. A risk score model of COAD immune genes was built. Through a series of clinical correlation analysis, it was found that 7 immune genes (SLC10A2, CXCL3, IGHV5-51, INHBA, STC1, UCN, and OXTR) were correlated with clinical prognosis and risk score of patients with COAD. These seven immune genes may provide potential therapeutic targets for clinical treatment of COAD.

## Data Availability

The data used to support the findings of this study are included within the article.

## Abbreviations

COAD: Colon adenocarcinoma
HR: Hazard ratio
TF: Transcription factor
Coef: Correlation coefficient of the gene
Std.err: Std. error difference
ID: Identity
cor: Correlation
LogFC: Log fold change
FDR: False discover rate
*P*: *P* value
Exp: Expression level of the gene
ROC: Receiver operating characteristic
K-M: Kaplan-Meier curve
